# Drivers and barriers of acceptance of eHealth interventions in postpartum mental health care: a cross-sectional study

**DOI:** 10.1186/s12889-025-25297-1

**Published:** 2025-11-17

**Authors:** Lisa Maria Jahre, Anna-Lena Frewer, Heidi Meyer, Katja Koelkebeck, Antonella Iannaccone, Eva-Maria Skoda, Martin Teufel, Alexander Bäuerle

**Affiliations:** 1 Clinic for Psychosomatic Medicine and Psychotherapy, LVR-University Hospital, University of Duisburg-Essen, Essen, Germany; 2 Center for Translational Neuro- and Behavioral Sciences (C-TNBS), University of Duisburg-Essen, Essen, Germany; 3 Department of Psychiatry and Psychotherapy, LVR-University Hospital Essen, University of Duisburg-Essen, Essen, Germany; 4 Department of Gynecology and Obstetrics, University Hospital Essen, Essen, Germany; 5 Department of Psychiatry and Psychotherapy, Bielefeld University, Medical School and University Medical Center OWL, Protestant Hospital of the Bethel Foundation, Bielefeld, Germany

**Keywords:** eHealth, Maternal mental health, Postpartum health, UTAUT, Women mental health

## Abstract

**Background:**

Postpartum mental health problems are common in women. Screening practice and treatment options are less common, which is a possible threat to health of mothers and children. eHealth interventions might bridge the gap but few validated programs are available. For developing relevant tools, an assessment of user behavior is a relevant step. Users’ acceptance of eHealth interventions can be examined via the Unified Theory of Acceptance and Use of Technology (UTAUT) model.

**Methods:**

A cross-sectional study was conducted between October 2022 and June 2023. Acceptance, sociodemographic, medical, psychometric, and eHealth data were assessed. This study included 453 women who had experienced pregnancy. Multiple hierarchical regression analysis and group comparisons (*t*-tests, ANOVA) were conducted.

**Results:**

High acceptance of eHealth interventions in postpartum mental health care was reported by 68.21% (*n* = 309) of women. Acceptance was significantly higher in women affected by mental illness, *t*(395) = -4.72, *p*_adj_ < 0.001, *d* = 0.50, and with postpartum depression (present or past), *t*(395) = -4.54, *p*_adj_ < 0.001, *d* = 0.46. Significant predictors of acceptance were Perceived support during pregnancy (β = − 0.15, *p* = .009), Quality of life (β = − 0.13, *p* = .022), Postpartum depression (β = 0.40, *p* = .001), Digital confidence (β = 0.18, *p* = .002), and the UTAUT predictors Effort expectancy (β = 0.10, *p* = .037), Performance expectancy (β = 0.50, *p* < .001) and Social influence (β = 0.25, *p* < .001). The extended UTAUT model was able to explain 59.82% of variance in acceptance.

**Conclusions:**

This study provides valuable insights into user behavior of women who experienced pregnancy. High acceptance towards eHealth interventions in postpartum mental health care and identified drivers and barriers should be taken into account when implementing tailored eHealth interventions for this vulnerable target group. Specifically women with mental health issues report high acceptance and should therefore be addressed in a targeted manner.

**Supplementary Information:**

The online version contains supplementary material available at 10.1186/s12889-025-25297-1.

## Background

Childbirth not only fundamentally effects the lives of the new parents, it also has a significant impact on the mental health of the childbearing mother. Psychological stress during pregnancy is thus common [1] and is, at the same time, a significant risk factor for postpartum mental illness [2]. The most common postpartum mental disorders are postpartum depression (PPD) and anxiety [3]. Whereas postpartum psychosis and post-traumatic stress disorder are less frequent, the severity of these disorders is highly relevant. In high-income countries, the point prevalence is 13% for PPD and around 10% for postpartum anxiety [3]. In the long term, burden of severe mental illness after childbirth, combined with other risk factors, may even lead to maternal suicide, which is described as the “leading cause of maternal death” [4]. PPD is also a crucial risk factor for negative outcomes in mothers as well as their children [5, 6], especially for maternal morbidity and maternal near miss [7]. Maternal mental health, quality of life and relationships are negatively impacted by PPD. Further, the infants’ development and mother-child interactions are complicated or impaired by PPD [5, 6]. It is evident that prevention and treatment of postpartum mental illness takes an important role in women’s care. Accordingly, the World Health Organization has developed recommendations for maternal and newborn care, which emphasizes the necessity of supportive, evidence-based mental health care after childbirth [8].

Early detection of PPD is essential for the implementation of appropriate treatment options [9]. However, screening rates for PPD are low [10]. There is no established routine screening program in Germany [11], even though validated instruments recommended by treatment guidelines are available [12]. Moreover, screening alone is not enough to alleviate the symptoms of those affected; appropriate treatment is required. PPD can be treated effectively with psychotherapy [13]. In Germany, however, psychotherapeutic care for people with PPD is not ensured in practice [14]. In addition to a lack of available treatment options, another access barrier is stigma around mental health [15]. This existing gap in maternal mental health care must be addressed with high priority in order to ensure the well-being of mothers and their children. eHealth (electronic health) may provide an evidence-based addition to established mental health treatments.

EHealth is a novel way to provide medical and psychological support services [16], and can be employed in different ways. For example, Internet-based cognitive behavioral therapy for depression has been proven to be as effective as face-to-face delivery [17]. Moreover, telehealth interventions are effective in decreasing symptoms of PPD, including anxiety and functional impairment [18–20]. Further, screening for PPD with eHealth tools seems to be a promising method, bypassing fears regarding openness about mental illness during the postpartum period [21]. On the other hand, there is a high number of available mHealth (mobile health) tools addressing postpartum mental health which are only of moderate quality and have not been clinically tested [22]. Users’ acceptance of such innovative eHealth services needs to be investigated in an evidence-based manner.

The successful implementation and patients’ adaption of eHealth services relies on the acceptance by its potential user base. According to the Unified Theory of Acceptance and Use of Technology (UTAUT), acceptance can be operationalized as behavioral intention to use such eHealth interventions [23, 24]. Acceptance is determined by three core factors: social influence (SI), performance expectancy (PE) and effort expectancy (EE). SI is conceptualized as the extent to which family or friends would approve of the use of a technology. PE stands for the expectation that the technology will lead to a beneficial effect for the user. EE describes the amount of effort a person expects to expend when using the technology [23, 24]. Acceptance of eHealth interventions has been investigated among different patient groups in a number of studies [25–28]. To this date, acceptance among women during the postpartum period has not been assessed. Further, studies suggest that, besides the three core predictors SI, PE and EE, additional factors should be integrated into the UTAUT model to obtain a full overview of relevant influences [23]. Therefore, it is important to examine the relevant drivers and barriers of eHealth use in postpartum mental health care. Only evidence-based evaluation of relevant factors influencing the use of eHealth interventions can ensure that effective health care services are actually adopted by their target group.

### Objectives

The aim of this study was to determine the drivers and barriers of acceptance of eHealth interventions in postpartum mental health care. Differences in acceptance based on sociodemographic, obstetric, psychometric and medical data were investigated with a cross-sectional study in a group of women who had experienced pregnancy.

## Methods

### Study design and study population

A cross-sectional, online-based questionnaire study was conducted to examine acceptance of eHealth interventions in postpartum mental health care among a convenience sample of women who had experienced pregnancy. Participants were recruited via flyers from the Clinic for Gynecology and Obstetrics of the University Hospital Essen, from gynecological outpatient practices in Essen, self-help groups and counselors specializing in postpartum mental illness in Germany, and from posts on social media channels centered on pregnancy and postpartum mental illness (Facebook, Instagram). Study information was presented in form of posters and flyers. Participation was anonymous and voluntary. Participants did not receive compensation. Inclusion criteria were legal age (≤ 18 years), female gender, history of pregnancy, sufficient command of the German language and Internet access. Exclusion criteria was missing data on the primary outcome (acceptance). There was no upper age limit to cover a diverse range of postpartum experiences. Data was collected between October 2022 and June 2023 via the platform Unipark [29]. Digital informed consent was given before the start of the survey. Average completion time was *M* = 16.63 (*SD* = 9.16) minutes. Initially, *N* = 558 participants started the questionnaire. *N* = 105 (18.82%) participants were excluded because they did not fulfill inclusion criteria or were missing data. Therefore, *N* = 453 participants (81.18%) were included in the final data analysis. The conductance of the study was approved by the Ethics Committee of the Medical Faculty of the University of Duisburg-Essen (19–89−47-BO).

### Assessment instruments

The survey encompassed sociodemographic, medical, psychometric, and eHealth data. Validated assessment instruments and self-generated items were used to acquire responses. The full questionnaire is provided in the Supplementary Materials.

Sociodemographic data included age, gender, marital status, educational level, occupational status and place of residence (population size). Medical data included self-reported presence of a somatic or mental illness and specifically the diagnosis of PPD.

Regarding obstetric data, participants were asked about number of pregnancies, number of children, age of children, time since childbirth, whether the pregnancy was planned and if their pregnancy was considered high-risk due to medical complication (e.g., preeclampsia, gestational diabetes or hypertension), as both aspects negatively affect postpartum mental health [30, 31].

Perceived support during the last pregnancy was rated on a scale from 0 to 10 (0 = “I did not feel supported at all”, 10 = “I felt extremely supported.”). Current quality of life was also indicated on a scale from 0 to 10 (0 = “very low quality of life”, 10 = “very high quality of life”).

Further, eHealth literacy was assessed using the revised German version of the eHealth Literacy Scale (GR-eHEALS; [32]). Responses were given on a five-point Likert scale (1 = “strongly disagree”, 5 = “strongly agree”), with higher scores indicating higher levels of eHealth literacy. The GR-eHEALS showed excellent validity in its original version [33] (Cronbach’s α = 0.88), and in the validated German translation [32] (Cronbach’s α = 0.83–0.92). Internal consistency in this study was excellent (Cronbach’s α = 0.92). Digital confidence [25, 28, 34] was assessed via three items (e.g., “How confident are you in using digital media?”) and responses were given on a five-point Likert scale (1 = “not very confident”, 5 = “very confident”), with higher scores indicating higher levels of digital confidence. Internal consistency was high (Cronbach’s α = 0.89). Internet anxiety [27, 34, 35] and digital overload [25, 27, 36] were also assessed via three items each and responses were given on a five-point Likert scale (e.g., “I have concerns about using the Internet.”, “I feel burdened by the constant accessibility via cell phone or e-mail.”, 1 = “strongly disagree”, 5 = “strongly agree”). Higher scores indicate higher levels of Internet anxiety and digital overload, respectively. Internal consistency was good (Cronbach’s α = 0.75 for Internet anxiety and α = 0.82 for digital overload).

In order to assess acceptance towards eHealth interventions in postpartum mental health care, an adapted version of the UTAUT model [23, 24] was applied. The UTAUT model is a validated model to assess and predict the acceptance of digital interventions [23]. Acceptance, which is operationalized as behavioral intention (BI), was determined by four items (e.g., “I would use such an eHealth intervention if it was offered to me.”). Internal consistency was high (Cronbach’s α = 0.86). Of the three core predictors of the UTAUT model, social influence (SI) was assessed via three items (e.g., “People close to me would support the use of such an eHealth intervention.”). Internal consistency was high (Cronbach’s α = 0.80). Performance expectancy (PE) consisted of four items (e.g., “Such an eHealth intervention could improve my overall well-being.”). Internal consistency was excellent (Cronbach’s α = 0.91). Three items were used to assess effort expectancy (EE; e.g. “Using such an eHealth intervention would not be an additional burden for me.”). Internal consistency was good (Cronbach’s α = 0.79). Responses for BI, SI, PE and EE were given on a five-point Likert scale (1 = “strongly disagree”, 5 = “strongly agree”), with higher scores indicating higher levels of BI, SI, PE, and EE.

### Statistical analysis

Statistical analysis was performed using R (4.3.8). Sum scores (GR-eHEALS) and mean scores (digital confidence, Internet anxiety, digital overload) were calculated. For acceptance (= BI) and its three predictors (EE, PE, SI) mean scores were calculated as well. Acceptance was further divided into categories, in accordance with prior research [25, 26, 34]: scores from 1 to 2.34 indicate low acceptance, scores from 2.35 to 3.67 indicate moderate acceptance and scores from 3.68 to 5 indicate high acceptance. Descriptive statistics were applied for sociodemographic, medical, obstetric, psychometric and eHealth data. Differences in acceptance based on educational level, somatic or mental illness, diagnosis of PPD, and risk during pregnancy were examined with independent *t*-tests and ANOVAs. *P*-values were adjusted for multiple comparisons via Bonferroni correction. Levene’s test indicated homoscedasticity. Due to the given sample size, normal distribution of residuals was assumed. Multiple hierarchical regression analysis was conducted to examine drivers and barriers of acceptance of eHealth interventions in postpartum mental health care. Predictors were included block wise: (1) sociodemographic and obstetric data, (2) medical and psychometric data, (3) eHealth data, (4) UTAUT predictors. The variance inflation factor (VIF) was used to verify the absence of multicollinearity (all VIF values < 2.0). Visual inspection of Q-Q-plots of the residuals showed no signs of violations against normality. Therefore, normal distribution of the residuals was assumed. Scatter plots of the standardized residuals and the adjusted predicted values verified homoscedasticity. The level of significance was set to α < 0.05 for all tests. Effect sizes were reported according to Cohen (1988 [37]), with values around 0.2, 0.5, and 0.8 indicating small, medium, and large effects, respectively.

## Results

### Study population

Among *n* = 453 female participants who experienced pregnancy in the past the mean age was *M* = 35.97 (*SD* = 6.55) years. The youngest participant was 18 years old and the oldest was 63 years old. The average number of pregnancies was *M* = 1.86 (*SD* = 1.18), while the number of children was *M* = 1.53 (*SD* = 0.76). The age of youngest child of the participants was *M* = 2.80 (*SD* = 5.00) years, and the oldest *M* = 4.51 (*SD* = 6.04) years. An average of *M* = 43.77 (*SD* = 63.70) months had passed since the last childbirth. A high-risk pregnancy in the past was reported of 31.79% (*n* = 144) of the participants. Among the participants, 79.47% (*n* = 360) stated that their last pregnancy was planned. On a scale from 0 to 10, participants reported an average perceived support during pregnancy of *M* = 6.97 (*SD* = 2.39) and their current quality of life of *M* = 7.10 (*SD* = 1.95).

In terms of eHealth, participants reported high eHealth literacy (*M* = 31.98, *SD* = 5.90, range 8–40) and high digital confidence (*M* = 4.20, *SD* = 0.79, range 1–5). Internet anxiety (*M* = 1.54, *SD* = 0.65, range 1–5) was low and digital overload was moderate (*M* = 2.70, *SD* = 1.03, range 1–5). Additional sample characteristics are given in Table 1.

**Table 1:** Sample characteristics

	*N* (%)
Marital status	
Single	21 (4.64)
In a relationship	79 (17.44)
Married	330 (72.85)
Divorced, separated	17 (3.75)
Other	6 (1.32)
Educational level	
No or lower secondary education/other	20 (4.42)
Higher secondary education	48 (10.60)
Higher education entrance qualification	114 (25.17)
University education	271 (59.82)
Occupational status	
Still in education, unemployed, unfit to work or other	30 (6.62)
House wife, parenting	21 (4.64)
Maternity leave	26 (5.74)
Parental leave	168 (37.09)
Part-time employed	143 (31.57)
Employed	64 (14.13)
Retired	1 (0.22)
Place of residence (population size)	
Large city (> 100,000 residents)	287 (63.36)
Medium sized city (> 20,000 residents)	78 (17.22)
Small town (> 5,000 residents)	34 (7.51)
Rural area (< 5,000 residents)	54 (11.92)
Somatic illness	
Yes	107 (23.62)
No	298 (65.78)
Not available	48 (10.60)
Mental illness	
Yes	137 (30.24)
No	260 (57.40)
Not available	56 (12.36)
Diagnosis of PPD	
Currently	10 (2.21)
In the past	46 (10.15)
Currently and in the past	12 (2.65)
No diagnosis, but suspected	102 (22.52)
No	227 (50.11)
Not available	56 (12.36)
Total	453 (100.00)

*PPD* Postpartum depression

### Acceptance of eHealth interventions in postpartum mental health care

Overall, acceptance of eHealth interventions in postpartum mental health care was high (*M* = 3.89, *SD* = 0.95, range 1–5). High acceptance was reported by 68.21% (*n* = 309) of the participants and 22.96% (*n* = 104) showed moderate acceptance, while only 8.83% (*n* = 40) participants gave responses that indicated low acceptance.

Acceptance of eHealth interventions in postpartum mental health care was significantly higher in mothers affected by mental illness, *t*(395) = −4.72, *p*_adj_ < 0.001, *d* = 0.50. Moreover, women who were currently suffering from postpartum depression or had been in the past, including those without diagnosis, reported a significantly higher level of acceptance compared to participants without depressive symptoms, *t*(395) = −4.54, *p*_adj_ < 0.001, *d* = 0.46. Differences in acceptance were not dependent on different levels of education, occurrence of a high-risk pregnancy or presence of a somatic disease, all *p*_adj_ >0.05.

### Predictors of acceptance of eHealth interventions in postpartum mental health care

Multiple hierarchical regression analysis was applied to determine predictors of acceptance of eHealth interventions in postpartum mental health care. Only complete cases were included in the analysis, which is based on the data of *n* = 327 participants.

First, sociodemographic and obstetric data were included (*R*^2^ = 0.024, *R*^2^_*adj*_ = 0.014, *F*(3,323) = 2.59, *p* =.053). *Perceived support during pregnancy* (β = − 0.15, *p* =.009) was a significant predictor of acceptance. The explained variance of the first step was 2.35%.

Psychometric and medical data, included in the second step (*R*^2^ = 0.086, *R*^2^_*adj*_ = 0.069, *F*(6,320) = 5.02, *p* <.001), significantly increased the explained variance to 8.60% (∆*R*^2^ = 0.062, *F*(3,320) = 16.23, *p* <.001). *Quality of life* (β = − 0.13, *p* =.022) and *PPD* (β = 0.40, *p* =.001) were revealed as significant predictors of acceptance.

In the third step, eHealth data (*R*^2^ = 0.123, *R*^2^_*adj*_ = 0.095, *F*(10,316) = 4.41, *p* <.001), significantly increased the explained variance to 12.26% (∆*R*^2^ = 0.037, *F*(4,316) = 7.12, *p* <.001). *Digital confidence* (β = 0.18, *p* =.002) was a significant predictor of acceptance.

In the final step, the three UTAUT predictors were included (*R*^2^ = 0.598, *R*^2^_*adj*_ = 0.582, *F*(13,313) = 35.85, *p* <.001). Explained variance of the final model was significantly increased to 59.82% (∆*R*^2^ = 0.475, *F*(3,313) = 123.53, *p* <.001). *EE* (β = 0.10, *p* =.037), *PE* (β = 0.50, *p* <.001) and *SI* (β = 0.25, *p* <.001) were significant predictors. Table 2 contains the final UTAUT model of acceptance and its predictors.

**Table 2:** Hierarchical regression model of acceptance of eHealth interventions in postpartum mental health care

Predictors	*B*	β	*t*	*R* ^2^	∆*R*^2^	*p*
(Intercept)	0.62	− 0.09	1.39			0.166
Step 1: Sociodemographic and obstetric data				0.024	0.024	
Age	− 0.00	− 0.03	−0.53			0.595
Time since birth	0.00	0.02	0.33			0.739
Perceived support during pregnancy	− 0.00	− 0.00	−0.07			0.942
Step 2: Psychometric and medical data				0.086	0.062	
Quality of life	− 0.04	− 0.09	−2.23			0.027
PPD	0.18	0.21	2.58			0.010
Somatic illness	− 0.00	− 0.00	−0.03			0.975
Step 3: eHealth data				0.123	0.037	
eHealth literacy	− 0.00	− 0.03	−0.76			0.448
Digital confidence	0.14	0.12	3.08			0.002
Internet anxiety	− 0.01	− 0.01	−0.15			0.877
Digital overload	0.02	0.02	0.46			0.643
Step 4: UTAUT predictors				0.598	0.475	
EE	0.10	0.09	2.10			0.037
PE	0.50	0.51	10.72			< 0.001
SI	0.25	0.23	5.47			< 0.001

*N* = 327. In Step 2, 3, and 4 only the newly included variables are presented

*B* Unstandardized beta, *β* Standardized beta, *t* Test statistic, *R²* Determination coefficient, *∆R*^*2*^ Changes in R^2^, *EE* Effort expectancy, *PE* Performance expectancy, *PHQ-8* Patient health questionnaire-8, *PPD* Postpartum depression, *SI* Social influence, *UTAUT* Unified theory of acceptance and use of technology

## Discussion

This study aimed to determine acceptance of eHealth interventions in postpartum mental health care, as well as its drivers and barriers. The strengths of this study were the direct involvement of women who had experienced pregnancy, and therefore the potential users of these eHealth offers, with the attention to their unique circumstances, challenges and needs, as well as the application of the validated, evidence-based UTAUT model. To our knowledge, the UTAUT model has not been applied in the context of postpartum mental health care and relevant factors influencing the use of eHealth services has not yet been explored in this sample.

Overall, women participating in this study reported a high level of acceptance of eHealth interventions in postpartum mental health care, with only a small number of participants indicating low levels of acceptance. Women with PPD or other mental illness in their medical history showed a higher level of acceptance than women who were not affected. There were no differences in acceptance based on level of education, occurrence of a high-risk pregnancy or diagnosis of a somatic disease. Significant predictors of acceptance were perceived support during pregnancy, quality of life, eHealth literacy, digital confidence, and the three core UTAUT predictors effort expectancy (EE), performance expectancy (PE), and social influence (SI). The extended UTAUT model, which included additional sociodemographic, medical, psychometric and eHealth characteristics, was able to explain a high level of variance in acceptance (59.82%).

The high level of acceptance found in the present study is comparable to the evidence of good acceptance of telehealth programs for high-risk pregnancy [38] and eHealth tools for gestational diabetes mellitus [39]. Only a very small proportion of participants showed low acceptance. These results provide a promising basis for the implementation of eHealth services in postpartum mental health care. On the other hand, obstacles to the actual use of such offers must be minimized and potential users with low acceptance should be specifically targeted to raise awareness and uptake of innovative eHealth interventions. Women may generally be more accepting of eHealth offers: In a cross-sectional study among cancer patients applying the UTAUT model, female gender was a significant predictor of higher levels of acceptance of video consultation in cancer care [34]. In addition, female patients affected by hypothyroidism also reported high levels of acceptance in a study determining the acceptance of mHealth services via the UTAUT model [40]. However, the evidence regarding gender differences in the context of eHealth is still inconclusive [34]. More research is therefore required to better understand the importance of gender for acceptance of eHealth.

Acceptance of eHealth interventions was higher among participants with PPD or with other mental illness. This finding is in line with research among patients affected by cardiac diseases [25] and obesity [41], which shows that patients affected by mental illness demonstrate greater acceptance of eHealth offers. One explanation for this may be the greater burden of suffering and, as a result, the greater need for immediate, easily accessible treatment services. In particular, mothers affected by PPD, who might be limited by child care or bed rest, would be able to receive adequate mental health care through eHealth interventions. As stigma of mental health issues, especially in the postpartum period, is an important barrier to help-seeking behavior [15], women affected by PPD or mental illness might prefer eHealth offers compared to face-to-face interventions because they expect less shame and fear of judgment by others in a digital treatment approach, which may offer more distant or even anonymous forms of support. Similarly, a higher quality of life predicted lower acceptance, which underlines the interpretation that less burdened individuals see no necessity in using eHealth interventions, while highly burdened individuals may especially profit from supplement eHealth offers.

Perceived social and medical support during pregnancy was negatively associated with acceptance of eHealth interventions in postpartum mental health care. A lack of social support during pregnancy is an important risk factor for PPD [42, 43]. It is plausible that women who already receive sufficient support during this unique phase of their lives have less need to use supportive eHealth services. In a study conducted in China, an app targeting women affected by PPD has proven to be effective in not only treating depressive symptoms but also increasing perceived social support [44]. eHealth offers may therefore especially support people who lack a strong social network in coping with the burden of PPD and to prevent severe consequences of PPD, such as maternal morbidity [7].

Higher digital confidence was related to higher acceptance of eHealth interventions. This finding is in line with previous research among patients with chronic pain [28, 45], cancer [34] and obesity [41]. Overall, digital confidence was high in this sample of young women. This association shows that people who feel comfortable using the Internet and use it frequently also report a higher level of openness towards eHealth. While highly confident Internet users may be easily approachable when implementing new eHealth interventions, those with lower confidence should be especially targeted. It might be helpful to offer specific support in the practical use of eHealth services to overcome this barrier.

Acceptance of eHealth interventions in postpartum mental health care was significantly predicted by the three UTAUT predictors EE, PE and SI, as reported in previous research among patients with post-COVID-19 syndrome [27], chronic pain [28, 45], cancer [34], obesity [41] and female patients with hypothyroidism [40]. EE describes the effort that a person has to invest in order to use eHealth [24]. PE comprises the expectation that the eHealth offer is able to positively influence general well-being, mental and physical health, and coping with psychological stress. SI, which conceptualizes the extent to which related parties, the general practitioner or the gynecologist would approve of the use of the eHealth offer. eHealth offers that are expected to involve minimal effort, offer a high benefit and receive positive approval from others are highly accepted by their users.

While sociodemographic, obstetric, psychometric, medical and eHealth characteristics only explained a small percentage of the variance in acceptance (12.26%), the inclusion of the three UTAUT core predictors substantially increased the explained variance to 59.82%. These results are in accordance with previous research [27, 28, 34, 40, 41], and indicate that women’s beliefs and attitudes towards the use of eHealth offers are more influential drivers in their acceptance of eHealth services than their sociodemographic background or medical history. This finding further highlights the relevance of the UTAUT framework as a valid model to investigate acceptance of eHealth technology.

### Clinical implications

The findings of this study provide an evidence-based foundation for the development and implementation of eHealth offers targeting postpartum mental health. The high level of acceptance, operationalized as behavioral intention, suggests that new or improved eHealth interventions in postpartum mental health care would reach a receptive group of potential users. Particularly vulnerable subgroups, such as women with mental illness (especially PPD), low support during pregnancy and reduced quality of life, would be promising target groups whose needs should be specifically addressed in eHealth interventions. By fostering digital confidence, acceptance of eHealth offers might be increased even further. In order to utilize the acceptance determining factors EE, PE and SI, eHealth offers should be designed in a way that simplifies their use, produces positive effects for their users and are well received by experts and relatives. Overall, factoring in acceptance offers the opportunity to enhance and improve treatment of postpartum mental health and to establish new forms of support.

### Strengths and limitations

The strengths of our study were the validated assessment of acceptance applying the adapted UTAUT model and the direct involvement of women who had experienced pregnancy, i.e., the potential future users of eHealth interventions in postpartum mental health care. Through this approach, the unique drivers and barriers of acceptance of this specific group could be identified and can be adequately addressed in future research and clinical practice.

The following limitations need to be considered when interpreting the results of the present study. Sociodemographic and medical data was based on self-report and could therefore not be objectively confirmed. Since the study was conducted online, sampling bias may have influenced the composition of the sample. The form of the questionnaire might have particularly appealed to people who are already online savvy and feel comfortable using digital media. People who dislike technology may therefore be underrepresented. Further, a sizable proportion of participants reported a high level of education. It is therefore important, especially in the context of eHealth offers, to address these subgroups in a particularly targeted manner. There was no limitation for participation regarding how much time has passed since pregnancy. Memory bias must therefore be taken into account. Further, postpartum experiences that occurred further in the past may differ from more recent ones, which might introduce variability to the findings that could obscure patterns relevant to younger postpartum women. On the other hand, looking back at a more distant postpartum period might offer some additional time of reflection on experiences and needs during this relevant time period. In regards to the UTAUT model, which operationalizes behavioral intention as acceptance, the intention-behavior gap needs to be considered [46, 47]. This concept describes the fact that not every intention is translated into action. Therefore, the actual usage behavior of specific eHealth intervention requires dedicated research.

## Conclusions

In conclusion, acceptance towards eHealth interventions in postpartum mental health care was high and relevant drivers and barriers such as mental illness, perceived support, digital confidence, and the UTAUT predictors could be revealed, highlighting the relevance of the UTAUT model for future research. Based on the findings of this study, efforts should be made to implement eHealth offers that bypass barriers to help seeking such as stigma, and complement and enhance existing routine care. Notably, the three UTAUT predictors effort expectancy, performance expectancy and social influence play a critical role in the acceptance of eHealth interventions and should therefore be specifically targeted in the development and implementation of eHealth offers. Mental health care in a particularly vulnerable and high-need group could thereby be improved by increasing acceptance and utilization.

## Supplementary Information

Supplementary Material 1.

## Data Availability

The datasets used and analyzed during the current study are available from the corresponding author on reasonable request.

## Abbreviations

EE: Effort expectancy
GR-eHEALS: Revised German version of the eHealth literacy scale
PE: Performance expectancy
PHQ-8: Patient health questionnaire-8
PPD: Postpartum depression
SI: Social influence
UTAUT: Unified theory of acceptance and use of technology
VIF: Variance inflation factor
