# Supplementary material for: Drivers and barriers of acceptance of eHealth interventions in postpartum mental health care: a cross-sectional study
Source: BMC Public Health. 2025 Nov 17;25:3966. doi: 10.1186/s12889-025-25297-1 (PMC12621414; doi:10.1186/s12889-025-25297-1)
Supplement: Supplementary file 1 — Supplementary Material 1. [file 12889_2025_25297_MOESM1_ESM.docx]

**- SUPPLEMENTARY MATERIALS -**

**Drivers and Barriers of Acceptance of eHealth Interventions
in Postpartum Mental Health Care: A Cross-Sectional Study**

Lisa Maria Jahre^1,2^, Anna-Lena Frewer^1,2^, Heidi Meyer^2,3^, Katja Koelkebeck^2,3,5^, Antonella Iannaccone^4^, Eva-Maria Skoda^1,2^, Martin Teufel ^1,2^, Alexander Bäuerle^1,2*^

^1^Clinic for Psychosomatic Medicine and Psychotherapy, LVR-University Hospital, University of Duisburg-Essen, Essen, Germany.

^2^Center for Translational Neuro- and Behavioral Sciences (C-TNBS), University of Duisburg-Essen, Essen, Germany.

^3^Department of Psychiatry and Psychotherapy, LVR-University Hospital Essen, University of Duisburg-Essen, Essen, Germany.

^4^Department of Gynecology and Obstetrics, University Hospital Essen, Essen, Germany.

^5^Department of Psychiatry and Psychotherapy. Bielefeld University, Medical School and University Medical Center OWL, Protestant Hospital of the Bethel Foundation, Bielefeld, Germany.

*Corresponding Author: Dr. Alexander Bäuerle (alexander.baeuerle@lvr.de)

# Study questionnaire

The questionnaire was originally administered in German and translated into English for publication of this study only.

| Variable | Item / method of assessment |
| --- | --- |
| **Sociodemographic data** |  |
| Age | Please indicate your age (in years): |
| Gender | Please indicate your gender:   - Male - Female - Non-binary |
| Marital status | Please indicate your marital status:   - Single - In a relationship - Married - Divorced / seperated - Other |
| Educational level | Please indicate your highest educational level:   - No or lower secondary education/other - Higher secondary education - Higher education entrance qualification - University education |
| Occupational status | Please indicate your current occupational status:   - Still in education, unemployed, unfit to work or other - House wife, parenting - Maternity leave - Parental leave - Part-time employed - Employed - Retired |
| Place of residence (population size) | Please indicate the place of residence you live in:   - Large city (> 100,000 residents) - Medium sized city (> 20,000 residents) - Small town (> 5,000 residents) - Rural area (< 5,000 residents) |
|  |  |
| **Medical data** |  |
| Somatic illness | Do you have a physical illness (diagnosed by a doctor)?   - No - Yes (please specify): |
| Mental illness | Do you have or have you had a mental illness (diagnosed by a doctor or psychotherapist)?   - No - Yes (please specify): |
| Diagnosis of postpartum depression | Have you ever been diagnosed with postpartum depression?   - Currently - In the past - Currently and in the past - No diagnosis, but suspected - No |
|  |  |
| **Obstetric data** |  |
| Number of pregnancies | How many times have you been pregnant?  Please specify the number: |
| Number of children | How many children do you have? |
| Age of children | Please indicate the age of your youngest child:  Please indicate the age of your oldest child: |
| Time since childbirth | Please select the date of your last childbirth: |
| Planned pregnancy | Was your last pregnancy planned?   - No - Yes |
| High-risk pregnancy | Did you have a high-risk pregnancy?  This also includes multiple pregnancies.   - No - Yes - I cannot say |
| Perceived support during the last pregnancy | On a scale from 0 to 10, how supported did you feel during your last pregnancy?   - 0 = I did not feel supported at all - 10 = I felt extremely supported |
|  |  |
| **Psychometric data** |  |
| Current quality of life | On a scale from 0 to 10, how would you rate your current quality of life?   - 0 = very low quality of life - 10 = very high quality of life |
|  |  |
| **eHealth data** |  |
| eHealth literacy | German version of the eHealth Literacy Scale  (GR-eHEALS; Marsall et al., 2022) |
| Digital confidence | How confident are you in using …  … digital media?  … Internet platforms?  … digital devices (e.g. computers)?   - 1 = not very confident, 5 = very confident   (Bäuerle et al., 2023; Nurtsch et al., 2024; Stoppok et al., 2022) |
| Internet anxiety | I have concerns about using the Internet.  I am afraid I might make an irreversible mistake when using the Internet.  The Internet is something that worries me.   - 1 = strongly disagree, 5 = strongly agree   (Nurtsch et al., 2024; Schröder et al., 2023; Zobeidi et al., 2023) |
| Digital overload | I feel burdened by the constant accessibility via cell phone or e-mail.  I feel bothered by unwanted messages and emails.  I feel uncomfortable carrying a mobile device all the time.   - 1 = strongly disagree, 5 = strongly agree   (Bäuerle et al., 2023; Rasool et al., 2022; Schröder et al., 2023) |
|  |  |
| **Unified Theory of Acceptance and Use of Technology (UTAUT)** | To what extent do each of the following statements regarding eHealth interventions in postpartum mental health care apply to you?   - 1 = strongly disagree, 5 = strongly agree   (Philippi et al., 2021; Venkatesh et al., 2003) |
| Acceptance towards eHealth interventions in postpartum mental health care  ( = *behavioral intention*) | I would like to try such an eHealth intervention.  I would use such an eHealth intervention if it was offered to me.  I would use such an eHealth intervention if my health insurance covered the costs.  I would recommend such an eHealth intervention to people I know. |
| *Social influence* | People close to me would support the use of such an eHealth intervention.  My general practitioner would support the use of such an eHealth intervention.  My gynaecologist would support the use of such an eHealth intervention. |
| *Performance expectancy* | Such an eHealth intervention could improve my overall well-being.  Such an eHealth intervention could help me to manage mental stress after giving birth.  Such an eHealth intervention could help me to improve my mental health after giving birth.  Such an eHealth intervention could help me to improve my physical health after giving birth. |
| *Effort expectancy* | Using such an eHealth intervention would not be an additional burden for me.  Such an eHealth intervention would be easy for me to use and understand.  I could incorporate the use of such an eHealth intervention into my everyday life. |

**References**

Bäuerle A, Mallien C, Rassaf T, Jahre L, Rammos C, Skoda EM, et al. Determining the Acceptance of Digital Cardiac Rehabilitation and Its Influencing Factors among Patients Affected by Cardiac Diseases. J Cardiovasc Dev Dis. 2023;10(4).

Marsall M, Engelmann G, Skoda EM, Teufel M, Bäuerle A. Measuring Electronic Health Literacy: Development, Validation, and Test of Measurement Invariance of a Revised German Version of the eHealth Literacy Scale. J Med Internet Res. 2022;24(2):e28252.

Nurtsch A, Teufel M, Jahre LM, Esber A, Rausch R, Tewes M, et al. Drivers and barriers of patients' acceptance of video consultation in cancer care. Digit Health. 2024;10:20552076231222108.

Philippi P, Baumeister H, Apolinario-Hagen J, Ebert DD, Hennemann S, Kott L, et al. Acceptance towards digital health interventions - Model validation and further development of the Unified Theory of Acceptance and Use of Technology. Internet Interv. 2021;26:100459.

Rasool T, Warraich NF, Sajid M. Examining the Impact of Technology Overload at the Workplace: A Systematic Review. SAGE Open. 2022;12(3).

Schröder J, Bäuerle A, Jahre LM, Skoda EM, Stettner M, Kleinschnitz C, et al. Acceptance, drivers, and barriers to use eHealth interventions in patients with post-COVID-19 syndrome for management of post-COVID-19 symptoms: a cross-sectional study. Ther Adv Neurol Disord. 2023;16:17562864231175730.

Stoppok P, Teufel M, Jahre L, Rometsch C, Mussgens D, Bingel U, et al. Determining the Influencing Factors on Acceptance of eHealth Pain Management Interventions Among Patients With Chronic Pain Using the Unified Theory of Acceptance and Use of Technology: Cross-sectional Study. JMIR Form Res. 2022;6(8):e37682.

Venkatesh, Morris, Davis, Davis. User Acceptance of Information Technology: Toward a Unified View. MIS Quarterly. 2003;27(3).

Zobeidi T, Homayoon SB, Yazdanpanah M, Komendantova N, Warner LA. Employing the TAM in predicting the use of online learning during and beyond the COVID-19 pandemic. Front Psychol. 2023;14:1104653.
